# Antiangiogenesis Roles of Exosomes with Fei-Liu-Ping Ointment Treatment are Involved in the Lung Carcinoma with the Lewis Xenograft Mouse Model

**DOI:** 10.1155/2020/9418593

**Published:** 2020-04-05

**Authors:** Qi Zheng, Haolong Liu, Huiting Fan, Ying Zhang, Wei Hou

**Affiliations:** ^1^Oncology Department, Guang'anmen Hospital, China Academy of Chinese Medical Sciences, Beijing 100053, China; ^2^Beijing Institute for Drug Control, NMPA Key Laboratory for Quality Evaluation of Traditional Chinese Medicine (Traditional Chinese Patent Medicine), Beijing Key Laboratory of Analysis and Evaluation on Chinese Medicine, Beijing 102206, China

## Abstract

Exosomes display efficient biocompatibility and represent valuable vehicles for drug or effective material delivery in a tumour-therapeutic approach. Following treatment with Fei-Liu-Ping (FLP) ointment, a traditional Chinese herbal formula, which is used for treating lung cancer patients, could inhibit lung carcinoma growth in clinical and animal studies. In the present study, the values of VEGF and PDGF, which were closely related to angiogenesis, were estimated in serum and carcinoma tissue exosomes to unveil the FLP effects on angiogenesis. The common inflammatory factors of IL-6, IL-1*β*, TNF-*α*, and TGF-*β* in serum exosomes were also detected with the Lewis xenograft model. *Methods*. Male C57BL/6 mice were randomly divided into four groups, namely, normal, model, cyclophosphamide (CTX), and FLP treatment groups. Histological structures were observed and imaged by H&E. CD31 expressions in tumour tissues were detected by immunofluorescence (IF) and western blot (WB). VEGF, PDGF, and PDGFR levels in exosomes, serum, tumour, and lung tissues were detected by enzyme-linked immunosorbent assay (ELISA), immunohistochemistry (IHC), and WB, respectively. IL-6, IL-1*β*, TNF-*α*, and TGF-*β* levels in exosomes were measured by multiplex immunoassay panels. *Results*. The results showed that FLP had tumour growth inhibition rate (39.31%). CD31 protein expression was obviously decreased in tumour tissues of CTX- and FLP-treated MO mice, compared to that of MO mice (*P* < 0.05 or *P* < 0.001). VEGF, PDGF, and PDGFR expression levels with FLP treatment were downregulated in exosomes, serum, tumour, and lung tissues compared to model group (*P* < 0.05 or *P* < 0.01). The expressions of IL-6, IL-1*β*, and TNF-*α* were downregulated in exosomes compared to the model group (*P* < 0.05 or *P* < 0.01). *Conclusions*. This study suggested that FLP had the ability of inhibiting tumourigenesis in a Lewis lung xenograft mouse model, whose therapeutic mechanisms might relate with the downregulation of angiogenesis factor and tumour inflammatory cytokines levels.

## 1. Introduction

Lung carcinoma is the most frequent lethal malignancy and the leading cause of cancer death worldwide, harbouring high incidence in Asian countries [1, 2]. Due to the lack of specific early symptoms, the patients with lung cancer are neglected in initial stage and are frequently diagnosed in an advanced stage. Management of lung cancer usually entails radiotherapy, chemotherapy, targeted therapy, and immunotherapy following surgery [3]; however, the five-year survival rate is only approximately 25–30% [4, 5]. And, thus, it is imperative to develop novel antitumour drugs or methods for alleviating or postponing the progress of lung cancer. Traditional Chinese medicine (TCM) has been shown to be effective in the treatment of lung cancer, especially in decreasing the risk of tumour recurrence and metastasis [6, 7]. Using systematic reviews and meta-analyses, scholars have recently reported that Chinese herbal medicine could improve survival, quality of life, and immune suppression and reduce tumour recurrence and metastasis in lung cancer patients by alleviating immune suppression [7, 8].

According to TCM theory, the basic pathogenesis of lung cancer involves the deficiency of qi and yin, damage of collaterals in lung, and turbid phlegm obstruction of lung. Therefore, a TCM formula named Fei-Liu-Ping (FLP) ointment (FLP ointment, currently named Yifei Qinghua granules), which was created by Prof. Bingkui Piao, has been used for more than 30 years [9]. FLP ointment is a yin-nourishing, toxin-removing, and blood-activating formula of TCM. Previous clinical studies have shown that FLP ointment possessed many marked anticancer properties, such as relieving the manifested symptoms, ameliorating the side effects of radiotherapy and chemotherapy, and reducing the dose of medication required, thus improving the quality of life in lung cancer patients [8–10]. FLP ointment is produced by the Beijing Huashen Pharmaceutical Co., LTD. as a hospital preparation (SFDA approval number Z20050851).

Although previous studies indicated that FLP ointment inhibits angiogenesis, the role of exosomes with FLP treatment, especially circulating exosomes in mediating tumour vasculogenesis, is unclear. To elucidate the mechanisms of FLP inhibiting vasculogenesis and to address the role circulating and carcinomatous tissue exosomes play, the values of vascular endothelial growth factor (VEGF), platelet-derived growth factor (PDGF), and PDGFR which were closely related to angiogenesis, were estimated in serum and carcinomatous tissue exosomes. The above proteins were also detected in carcinomatous tissue. Common inflammatory factors, such as IL-6, IL-1*β*, TNF-*α*, and TGF-*β* in serum exosomes, were also measured in this study.

Extracellular microenvironment around lung carcinoma can contribute to the increase in the tumour metastasis and the grade malignancy. Exosomes are lipid-bilayer membrane vehicles that range from 30 to 100 nm in size and can be released into extracellular microenvironment by almost all cell types [11]. Although exosomes were previously considered as dumped vehicles from adjacent cells, their intercellular communication abilities have been proved, especially in carcinoma [12]. In the past decade, extracellular vesicles including apoptotic small body, microvesicles, and exosomes emerged as critical players in cell to cell communications in physiology and pathology [13]. Exosomes are tens nm in size, which allows genetic and molecular exchanges, including the transfer of proteins such as VEGF, PDGF, and PDGFR at a distance to target cells.

## 2. Materials and Methods

### 2.1. Reagents

FLP ointment (No. Z20063236) was prepared by the Pharmaceutical Preparation Centre of Guang'anmen Hospital and the China Academy of Chinese Medical Sciences (batch number 20171211). Voucher specimens were deposited at the School of Traditional Chinese Medicine at Capital Medical University in China. Cyclophosphamide (CTX, No. H32020857) was supplied by Jiangsu Sheng Di Pharmaceutics Co. Ltd. (Jiangsu, China). Dulbecco's modified Eagle's medium (DME H-21 4.5 g/Litre glucose), foetal bovine serum (FBS), penicillin, and streptomycin were purchased from Gibco Co. Ltd. (Grand Island, NY, USA). Rabbit anti-CD31 antibody, rabbit anti-VEGF antibody, and rabbit anti-PDGF antibody were purchased from Abcam (Cambridge, MA, UK). CD63 (H-193) rabbit polyclonal IgG antibody was purchased from Santa Cruz Biotechnology (Santa Cruz, CA, USA). Rabbit anti-Alix (3A9) antibody and rabbit anti-PDGFR antibody were purchased from Cell Signaling Technology (Danvers, MA, USA). The sheep anti-rabbit-FITC secondary antibody and DAPI were purchased from Beijing Biosynthesis Biotechnology Co. Ltd. (Beijing, China). VEGF, PDGF, and PDGFR enzyme-linked immunosorbent assay (ELISA) kits were purchased from Millipore Co. Ltd. (Billerica, MA, USA). A ProcartaPlex mouse basic kits for IL-6, IL-1*β*, TNF-*α*, and TGF-*β* were purchased from Thermo Fisher Scientific (Waltham, MA, USA). Western blot (WB) kits were supplied by Applygen Technologies Inc. (Beijing, China). All samples were identified by Associate Professor Rong Luo from the School of Traditional Chinese Medicine, Capital Medical University (Beijing, China). The voucher specimens of all samples were stored at Oncology Department, Guang'anmen Hospital, China Academy of Chinese Medical Sciences (Beijing, China).

#### 2.1.1. Preparation of FLP Ointment

FLP ointment was composed of several kinds of herbal medicines (Table 1). Briefly, all herbs were provided by the Guang'anmen Hospital and decocted twice with eightfold volume of distilled water for 1 h. The decoction was collected, filtered, merged, concentrated to 2 g/mL (equivalent to crude herb materials), and stored at 4°C for oral use. To ensure the quality and stability of FLP ointment, ultra-high-performance liquid chromatography combined with LTQ-Orbitrap mass spectrometry (UHPLC-Orbitrap MS, Thermo Fisher Scientific, San Jose, USA) was used to identify the active ingredients.

#### 2.1.2. UHPLC-Orbitrap MS Analysis of FLP Ointment

**Figure 1:**
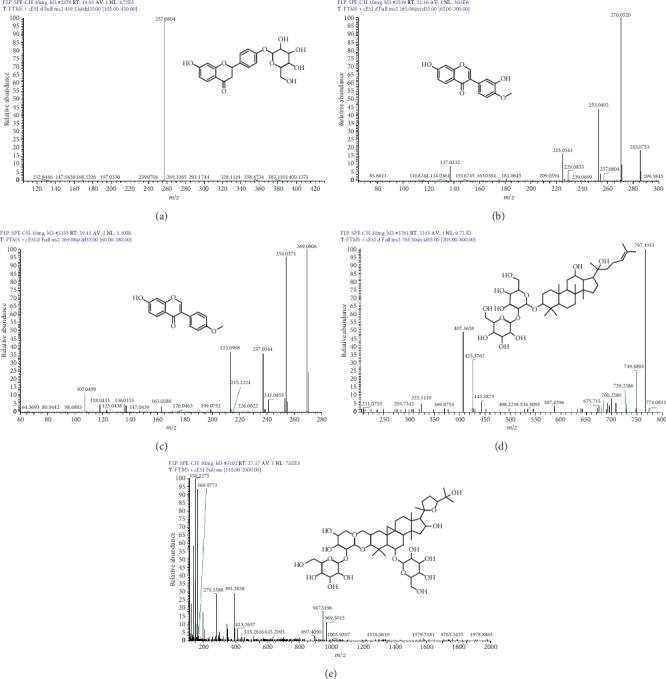
The chemical structures and Mass Spectrogram of (a) liquiritin, (b) calycosin, (c) formononetin, (d) ginsenoside Rg3, and (e) astragaloside VI, which may have pharmacoactivity of FLP ointment.

**Figure 2:**
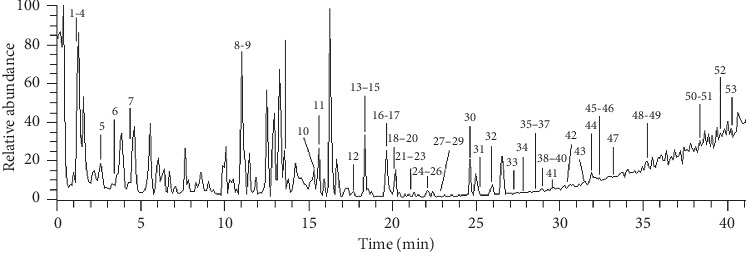
HPLC chromatogram of FLP ointment.

The analysis of the extract was performed with a UHPLC-Orbitrap MS with a Thermo BDS HYPERSIL C18 column (2.1 mm × 150 mm, ID 3 *μ*m). The column temperature was set at 30°C. The mobile phase was water (0.1% formic acid, phase A) and acetonitrile (phase B) with a gradient programme as follows: 0.0–25.0 min, 5%–30% B; 25.0–35.0 min, 30%–55% B; 35.0–40.0 min, 55%–75% B; 40.0–45.0 min, 75%–90% B; and 45.0–50.0 min, 90% B. The flow rate was 0.3 ml/min. All solvents were filtered through a 0.45 *μ*m filter before use. The injection volume was 10 *μ*L. The MS spectra were acquired in the positive ion mode with an electrospray ionization source (ESI+). The ESI source conditions were as follows: heater temp 350°C; sheath flow rate 35 arb; aux gas flow rate 10 arb; spray voltage 3.5 kV; capillary temp 350°C; and resolution 30000. Full-scan spectra were acquired in the mass range of *m*/*z* 50–1000. The main chemical structures and Mass Spectrogram which may have pharmacoactivity of FLP ointment were shown in Figure 1. Components of FLP ointment identified and HPLC chromatogram were shown in Table 2 and Figure 2, respectively. Additionally, the fragment ions (*m*/*z*) identification of compounds was shown in the supplemental files.

### 2.2. Experimental Animals

Male C57BL/6 mice (aged 6–8 weeks; weight 18.0–20.0 g) were purchased from Beijing Weitong Lihua Experimental Animal Tech. Co., Ltd., China [certification No. SCXK (JING) 2016–0011]. The mice were housed at the China Academy of Chinese Medical Sciences. The mice were housed under a 12-hour light/dark cycle in individual ventilated cages and maintained in a specific pathogen-free grade environment. All the experiments were approved and regulated by the Ethical Committee of the China Academy of Chinese Medical Sciences.

#### 2.2.1. Cell Culture

Lewis lung carcinoma cells were purchased from the National Infrastructure of Cell Line Resource and were cultured in Dulbecco's modified Eagle's medium (DME H-21 4.5 g/Litre glucose) containing 10% foetal bovine serum (FBS), 100 U/ml penicillin, and 100 mg/ml streptomycin in a cell culture incubator at 37°C under 5% CO_2_. Cells were collected at the logarithmic phase of growth by treatment with 0.25% trypsin for 2 min, and then the cell concentration was adjusted to 1 × 10^6^ with phosphate buffered saline (PBS). A Trypan blue exclusion test indicated that the number of living cells was greater than 95%.

### 2.3. Model Establishment and Experimental Treatment

After 5 days of acclimation, the mice were randomly divided into 4 groups: the normal control (NC), model (MO), CTX-treated MO, and FLP-treated MO groups (*n* = 10 mice per group). 12 g/kg/day dose of FLP ointment used in the present study was shown to have more antitumour effects in Lewis lung carcinoma xenograft mouse in our previous study [14, 15]. A subcutaneous injection suspended in 0.2 mL PBS with 2 × 10^5^ Lewis cells was implanted into the right flank of each MO mouse. The mice in the CTX-treated MO group were treated with 60 mg/kg CTX 24 h after the subcutaneous injection of the Lewis cells, as described below. The mice in the FLP-treated MO group were treated with suspensions of 12 g/kg/day FLP by gavage. The mice in the NC and MO groups were treated with normal saline. The CTX mice were injected only once, and the NC, MO, and FLP-treated mice were treated once per day for 21 days.

### 2.4. Sample Collection

The mice were sacrificed on day 21. Briefly, peripheral blood was collected via retro-orbital eye bleeds. Serum from each mouse was collected into 1.5 ml sterilised micro-tubes, kept on ice for 2 h, centrifuged at 4°C at 3000 rpm for 15 min, and stored at −80°C until further analysis. The lung and tumour tissues were immediately frozen for WB analysis, and 4% paraformaldehyde was used to fix the lung and tumour tissues for haematoxylin-eosin (H&E) staining, immunofluorescence (IF), and immunohistochemical (IHC) analysis. The volume of tumours was measured daily from day 8 until the mice were sacrificed and the tumour volume was calculated using the formula *π* *∗* (length) *∗* (width)^2^/6.

### 2.5. Tumour Inhibition Rate

Mice were sacrificed by cervical dislocation on day 21, and then the tumour tissues were harvested and weighed. The tumour inhibition rate was calculated as follows: tumour inhibition rate (%) = (1 – average weight of tumours in treatment group/average weight of tumours in control group) × 100.

### 2.6. Exosome Isolation

Exosomes were isolated from the serum of the individual groups of mice according to the manufacturers' instructions. ExoQuick Exosome Precipitation Solution (63 *μ*l) was added to 250 *μ*l serum and then refrigerated for 30 min. ExoQuick/serum mixtures were centrifuged at 1500 ×*g* for 30 min. All traces of fluid were removed by aspiration. Exosome pellets were resuspended in 30 *μ*l nuclease-free water and stored at −80°C until further analysis.

### 2.7. Histopathology

Mouse tumour tissues were dissected from the individual groups of mice and immediately fixed in 4% paraformaldehyde. The paraffin-embedded tumour tissue sections (5–10 *μ*m) were stained with H&E and then examined under a light microscope.

### 2.8. IF Staining and Analysis

Samples of tumours were prepared after fixation in 4% paraformaldehyde and embedded in paraffin. The slides were incubated with primary antibodies [rabbit anti-mouse CD31 (1 : 100)] at 4°C for 24 h. Subsequently, the slices were washed 3 times with PBS, incubated with the secondary antibody (sheep anti-rabbit-FITC, 1 : 800) at 37°C for 60 min, and then washed 3 times with PBS. The slices were counterstained with DAPI and maintained at 4°C. Finally, the slices were dehydrated and mounted for microscopic observation (Leica Microsystems, Wetzlar, Germany).

### 2.9. ELISA Detection

ELISA analysis was performed according to the manufacturers' instructions and analysed using a spectrometer at an absorbance of 450 nm, which was used as the reference wavelength (Synogen4, Gene Company Ltd.).

### 2.10. IHC Staining and Analysis

Samples of lungs and tumours were prepared after fixation in 4% paraformaldehyde and embedded in paraffin. The slides were incubated with primary antibodies [rabbit anti-VEGF (1 : 200), rabbit anti-PDGF (1 : 200), and rabbit anti-PDGFR (1 : 200)] at 4°C for 24 h. Subsequently, the sections were incubated with the biotin-labelled secondary antibody (sheep anti-rabbit IgG) at 37°C for 60 min. Colour development was accomplished by exposure to 3,3′-diaminobenzidinetrahydrochloride (DAB) for 40 sec to 1 min. Finally, the sections were dehydrated and mounted for microscopic observation. Quantitative analysis of the immunohistochemically stained images was carried out with a NIS-Elements BR 3.0 system. Three to five high-power fields (×400) were selected from four sections of the lateral ventricle in each group, and positive results were expressed as IOD values.

### 2.11. WB Analysis

The exosome proteins were extracted after homogenization with protein extraction agent (50 mmol/L Tris-HCl, pH 7.4; 10% SDS; 1 mmol/L PMSF, 0.25% deoxycholic acid sodium) for 30 min on ice. The samples were then centrifuged at 12,000 rpm at 4°C for 10 min, supernatants were collected, and protein concentrations were measured by a BCA assay. The lung and tumour proteins were extracted and measured as described above. Protein bands were separated by SDS-polyacrylamide gel electrophoresis (PAGE) and subsequently transferred onto nitrocellulose membranes (Millipore). Membranes were probed with primary rabbit anti-CD63 (1 : 2,000), rabbit anti-Alix (1 : 2,000) antibody, rabbit anti-CD31 (1 : 5,000) antibody, rabbit anti-VEGF antibody (1 : 5,000), rabbit anti-PDGF antibody (1 : 5,000), rabbit anti-PDGFR antibody (1 : 5,000), rabbit polyclonal anti-GAPDH antibody (1 : 10,000), or rabbit polyclonal anti-actin antibody (1 : 10,000) in blocking solution at 4°C overnight. Then, the membranes were incubated with secondary goat anti-rabbit IgG (1 : 7,000) for 60 min and electrochemiluminescence (ECL) reagent for 30 sec to 2 min. The membranes were exposed to X-ray film and photographed with a BIO-RAD ChemiDoc XRS gel imaging system. The pictures were exported by using Quantity One software and were analysed by using Image-Pro Plus 4.5 software (Media Cybernetics, Bethesda, MD, USA).

### 2.12. ProcartaPlex Immunoassays

ProcartaPlex multifactor detection was performed using magnetic beads that could be measured by means of fluorescent staining. The magnetic beads for different detection substances were mixed, and then the samples were added. The capture molecules and the target molecules were specifically bound in the suspension. The plates were read by a Luminex instrument for classification and quantification.

### 2.13. Statistical Analysis

The data were expressed as the mean standard error (SE) and were analysed with SPSS version 21.0 (SPSS Inc., Chicago, IL, USA). The test of normality was conducted by one-way ANOVA with a post hoc LSD test; otherwise, a rank-sum test was performed. A value of *P* < 0.05 was considered to indicate statistical significance.

## 3. Results

### 3.1. The Tumour Volume of Mice with a Lewis Lung Xenograft

**Figure 3:**
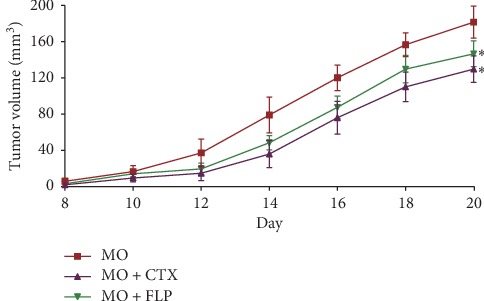
The tumour volume of mice in the Lewis lung xenograft group. Data are represented as the mean ± SEM. ^*∗*^*P* < 0.05 vs. MO.

The effect of FLP ointment was tested in a Lewis lung xenograft mouse model generated by Lewis cell line. The mice were treated for 21 days from the day Lewis cells were implanted in all MO mice. The volume of the tumours was measured daily from day 8 until the mice were sacrificed. On day 21, the mice injected with Lewis cell were sacrificed; the mean volume of the tumours in the CTX-treated group was 130.28 ± 14.82 mm^3^ and in the FLP-treated group was 147.21 ± 14.33 mm^3^, significantly lower than that in the control group which was 182.28 ± 17.72 mm^3^ (*P* < 0.05, Figure 3).

### 3.2. Treatment with FLP Ointment Inhibits Tumour Growth of Lewis Lung Cancer Xenografted Mice

**Figure 4:**
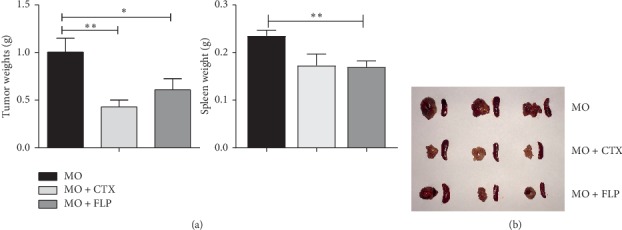
The tumour and spleen weights of mice in the Lewis lung xenograft group. The mice were sacrificed on day 21, and tumour and spleen tissues were measured and weighed. (a) The average tumour weights of MO, MO + CTX, and MO + FLP mice were 1.00 ± 0.15 g, 0.43 ± 0.07 g, and 0.61 ± 0.11 g. The spleen weights of MO, MO + CTX, and MO + FLP mice were 0.24 ± 0.01 g, 0.17 ± 0.02 g, and 0.17 ± 0.01 g. Data are represented as the mean ± SEM. ^*∗*^*P* < 0.05, ^*∗∗*^*P* < 0.01 vs. MO. (b) Tumours and spleens in the MO, CTX-treated, and FLP-treated groups.

The mice were sacrificed on day 21, and tumour and spleen tissues were measured and weighed. The average tumour weight of MO mice was 1.00 ± 0.15 g. Compared to that of MO mice, the average tumour weight was significantly lower in FLP-treated and CTX-treated mice (*P* < 0.05, *P* < 0.01; Figure 4(a)). The average spleen weight of MO mice was 0.24 ± 0.01 g. Compared to that of MO mice, the average spleen weight was significantly lower in FLP-treated mice (*P* < 0.05; Figure 4(a)). The FLP inhibition rate was 39.31%, and the CTX inhibition rate was 57.29%. Based on the abovementioned results, we found that FLP ointment could inhibit the tumour growth of Lewis lung cancer cells.

### 3.3. Pathological Changes in Tumour Tissues

**Figure 5:**
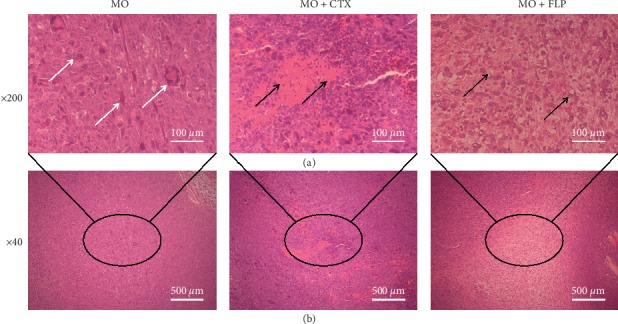
Observation of pathological changes in the tumours of mice under light microscopy. Magnification: ×200 (a) and ×40 (b). The histological changes were shown in the tumours of mice in the MO-, CTX-, and FLP-treated groups on day 21, respectively. The white arrows in the figure represent cellular atypia structures and black arrows represent necrotic cells. MO mice showed large tumour cells that were arranged in disordered structures and a large number of cellular atypia structures, while CTX-treated MO mice showed a larger number of necrotic cells, and FLP-treated MO mice exhibited sporadic necrotic cells; cell membranes dissolved and the nucleolus disappeared.

The pathological changes in the tumours of mice were observed by light microscopic evaluation of H&E-stained tissues. The tumour cells were large and arranged in a disordered manner. The nuclei were intensely stained, and most of the cells were divided in the MO group. In addition, MO mice showed large tumour cells that were arranged in disordered structures and a large number of cellular atypia structures. Conversely, treatment with CTX increased the number of necrotic cells. FLP-treated MO mice exhibited sporadic necrotic cells, cell membranes dissolved, and the nucleolus disappeared (Figure 5).

### 3.4. IF and WB Analysis of CD31 Proteins in Tumour Tissues

**Figure 6:**
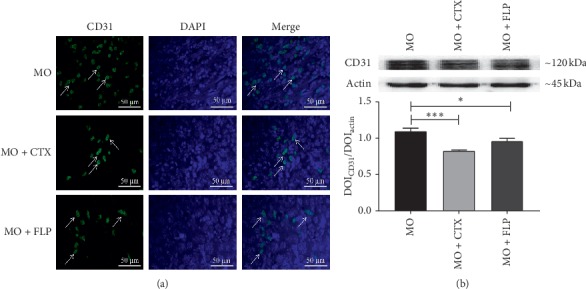
IF and WB analysis of the effect of FLP ointment on CD31 protein expression in tumour tissues of mice. (a) The fluorescence picture showed the high expression of CD31 in the tumours of MO mice, while CTX- and FLP-treated MO mice were reduced. (b) WB results showed that CD31 protein expression was obviously decreased in tumour tissues of CTX- and FLP-treated MO mice, compared to that of MO mice. Data are represented as the mean ± SEM. ^*∗*^*P* < 0.05, ^*∗∗∗*^*P* < 0.001 vs. MO.

Microangiogenesis plays an important role in tumour growth and angiogenesis. CD31 was expressed in vascular endothelial cells, which could be considered as a potential marker of microangiogenesis. The expressions of CD31 in tumour tissues were detected by IF and WB. The fluorescence picture showed the high expression of CD31 in the tumours of MO mice. And when the CTX- and FLP-treated MO mice were observed, the expressions were significantly reduced. For the further study, we detected CD31 by WB. Results showed that CD31 protein expression was obviously decreased in tumour tissues of CTX- and FLP-treated MO mice, compared to that of MO mice (*P* < 0.001 and *P* < 0.05, respectively, Figure 6).

### 3.5. WB Analysis of CD63 and Alix Proteins in Serum Exosomes

**Figure 7:**
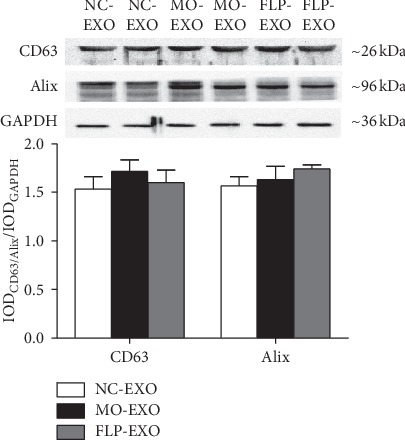
WB analysis of the effect of FLP ointment on CD63 and Alix protein expression in the serum exosomes of mice. Data are represented as the mean ± SEM.

Exosomes are a type of membranous vesicles that form from late endosomal or cytosolic budding. Exosomes usually carry proteins and miRNAs from the parent cells. As the marker proteins of exosomes, CD63 and Alix proteins were analysed by WB. The results showed which proteins the NC-EXO, MO-EXO, and FLP-EXO expressed. Compared to that of NC mice, CD63 and Alix protein expressions were increased in model mice and FLP mice (Figure 7). No significant correlation of CD63 and Alix protein expression levels were detected between MO and FLP mice.

### 3.6. ELISA Analysis of VEGF, PDGF, and PDGFR in Serum and Serum Exosomes

Tumour growth, invasion, and metastasis rely on angiogenesis. VEGF is a crucial angiogenic growth factor, and its level is a critical marker of angiogenesis. The PDGF/PDGF receptor signalling pathway plays a pivotal role in angioblast differentiation and tube formation; therefore, the expression of VEGF, PDGF, and PDGFR was analysed.

**Figure 8:**
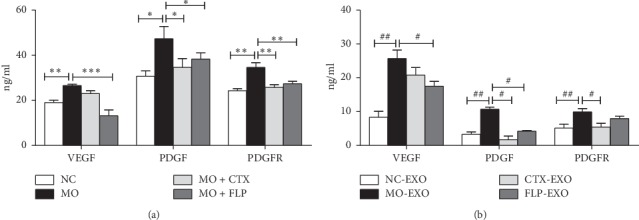
ELISA analysis of the effect of FLP ointment on VEGF, PDGF, and PDGFR protein expression in the serum and serum exosomes of mice. (a) The expression of VEGF, PDGF, and PDGFR in the serum. (b) The expression of VEGF, PDGF, and PDGFR in the serum exosomes. Data are represented as the mean ± SEM. ^*∗*^*P* < 0.05, ^*∗∗*^*P* < 0.01, ^*∗∗∗*^*P* < 0.001 vs. MO; ^#^*P* < 0.05, ^##^*P* < 0.01 vs. MO-EXOs.

ELISA analysis demonstrated that VEGF, PDGF, and PDGFR protein expression in serum were significantly increased in MO mice compared to those of NC mice (*P* < 0.01 and *P* < 0.05, respectively). In contrast, the expression levels of these proteins were significantly reduced in serum in FLP-treated MO mice compared to those of MO mice (*P* < 0.001, *P* < 0.01 and *P* < 0.05, respectively). In addition, compared to those of MO mice, PDGF and PDGFR protein expression were obviously decreased in the serum of CTX-treated MO mice (*P* < 0.01 and *P* < 0.05, respectively, Figure 8(a)).

VEGF, PDGF, and PDGFR protein expression were increased significantly in serum exosomes of MO mice compared to those of NC mice (*P* < 0.01). Additionally, VEGF and PDGF protein levels were significantly decreased in FLP-treated MO mice compared to those of MO mice (*P* < 0.05). CTX significantly decreased PDGF and PDGFR protein in serum exosomes of MO mice compared to those of MO mice (*P* < 0.05, Figure 8(b)).

### 3.7. IHC Analysis of VEGF, PDGF, and PDGFR in Lung and Tumour Tissues

**Figure 9:**
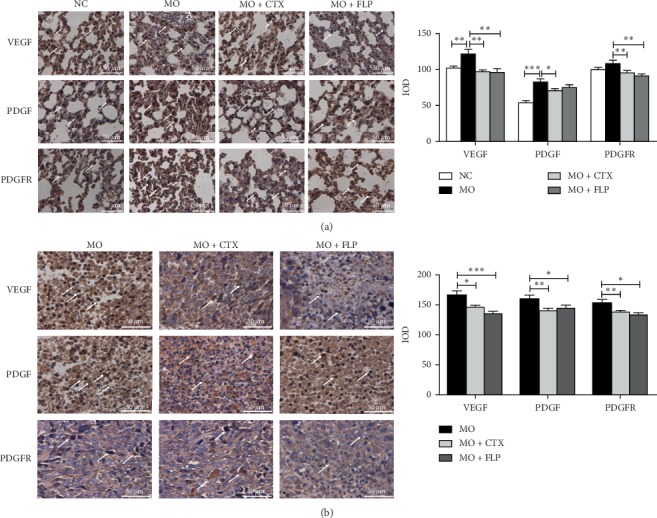
IHC analysis of the effect of FLP ointment on VEGF, PDGF, and PDGFR protein expression in lung and tumour tissues of mice. (a) The expression of VEGF, PDGF, and PDGFR in the lung. (b) The expression of VEGF, PDGF, and PDGFR in the tumour tissues. Data are represented as the mean ± SEM. ^*∗*^*P* < 0.05, ^*∗∗*^*P* < 0.01, ^*∗∗∗*^*P* < 0.001 vs. MO.

To further confirm the FLP ointment functions, IHC experiments were carried out. VEGF, PDGF, and PDGFR protein expression were analysed with IHC analysis. The results showed that VEGF and PDGF protein expression were significantly increased in the lungs of MO mice compared to those of NC mice (*P* < 0.01 and *P* < 0.001, Figure 9(a)). Treatment with CTX significantly decreased the VEGF, PDGF, and PDGFR levels in the lung compared to those of MO mice (*P* < 0.05 and *P* < 0.01, Figure 8(a)). VEGF and PDGFR protein levels in lung were significantly decreased in FLP-treated MO mice compared to those of MO mice (*P* < 0.01, Figure 9(a)). In addition, compared to those of MO mice, VEGF, PDGF, and PDGFR protein expression were obviously decreased in tumour tissues of CTX-treated and FLP-treated MO mice (*P* < 0.001, *P* < 0.01 and *P* < 0.05, respectively Figure 9(b)).

### 3.8. WB Analysis of VEGF, PDGF, and PDGFR in Lung and Tumour Tissues

**Figure 10:**
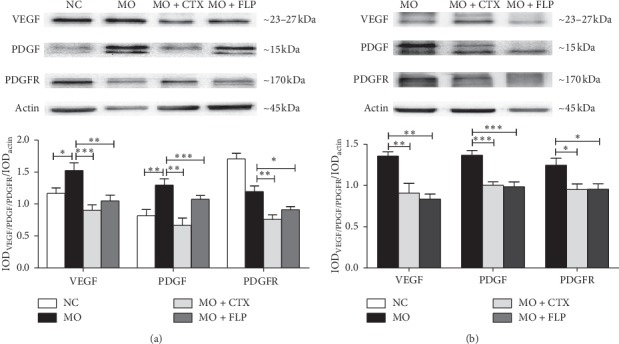
WB analysis of the effect of FLP ointment on VEGF, PDGF, and PDGFR protein expression in lung and tumour tissues of mice. (a) The expression of VEGF, PDGF, and PDGFR in the lung. (b) The expression of VEGF, PDGF, and PDGFR in the tumour tissues. Data are represented as the mean ± SEM. ^*∗*^*P* < 0.05, ^*∗∗*^*P* < 0.01, ^*∗∗∗*^*P* < 0.001 vs. MO.

Moreover, VEGF, PDGF, and PDGFR protein expression were analysed with WB. The results showed that VEGF and PDGF protein expression were significantly increased in the lungs of MO mice compared to those of NC mice (*P* < 0.05 and *P* < 0.01, Figure 10(a)). Treatment with CTX or FLP significantly decreased VEGF, PDGF, and PDGFR levels in lung and tumour tissues compared to those of MO mice (*P* < 0.001, *P* < 0.01 and *P* < 0.05, Figures 10(a) and 10(b)).

### 3.9. ProcartaPlex Immunoassay Analysis of IL-6, IL-1*β*, and TNF-*α* in Serum Exosomes and Tumours

**Figure 11:**
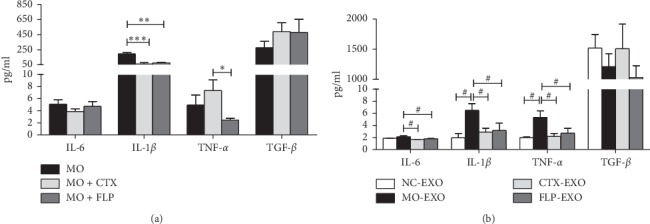
ProcartaPlex immunoassay analysis of the effect of FLP ointment on IL-6, IL-1*β*, TNF-*α*, and TGF-*β* levels in the serum exosomes and tumours of mice. (a) Changes in cytokines in the tumours of Lewis lung carcinoma xenografted mice. (b) Changes in cytokines in the serum exosomes of Lewis lung carcinoma xenografted mice. Data are represented as the mean ± SEM. ^*∗*^*P* < 0.05, ^*∗∗*^*P* < 0.01, ^*∗∗∗*^*P* < 0.001 vs. MO; ^#^*P* < 0.05 vs. MO-EXO.

Compared to that of MO mice, the level of IL-1*β* was significantly decreased in the tumours of CTX- and FLP-treated MO mice (*P* < 0.001 and *P* < 0.01, respectively). TNF-*α* levels did not exhibit obvious differences in the tumours of MO mice compared to those of CTX-treated mice; however, TNF-*α* levels were obviously decreased in FLP-treated MO mice compared to those of CTX-treated mice (*P* < 0.05). There were no significant differences in the IL-6 levels among the 3 groups (Figure 11(a)).

Compared to those of NC mice, the levels of IL-6, IL-1*β*, and TNF-*α* were decreased in the serum exosomes of MO mice. The IL-1*β* and TNF-*α* levels were markedly increased in serum exosomes of MO mice compared to those of NC mice (*P* < 0.05). Additionally, IL-1*β* and TNF-*α* expression were significantly decreased in CTX-treated and FLP-treated MO mice compared to those of MO mice (*P* < 0.05). FLP ointment significantly decreased IL-6 levels in serum exosomes of MO mice compared to those of untreated MO mice (*P* < 0.05, Figure 11(b)).

## 4. Discussion

The development of antitumour medicine is a hot field all the time in therapeutic tumour strategy in the last decades [16]. Even though many targeted antitumour medicines have been applied to clinic, the resistance during taking medicine remains to be the critical factor of chemistry therapy failure. And postsurgical adjuvant chemotherapy is commonly used to reduce the risk of lung carcinoma recurrence. However, the biological heterogeneities in lung carcinoma reduce the chemotherapeutic efficiency. FLP ointment, a traditional Chinese herbal formula, could inhibit lung cancer invasion by regulating the tumour inflammatory microenvironment through the NF-*κ*B signalling pathway in the previous study [14]. In combination with celecoxib, FLP ointment enhanced the antitumour growth and antimetastatic effects and may have been associated with the cyclooxygenase (Cox)-2 pathway [15].

The results of this study showed that the tumours of Lewis lung carcinoma xenografted mice were pathologically characterised by large cells with a disordered arrangement, shrinkage and fission, and nuclei that were strongly stained and lysogenic, which is consistent with previous reports [14, 15]. Compared with Lewis lung carcinoma xenografted mice, mice treated with FLP ointment manifested reduced tumour weight, and increased necrotic cells in the tumours. Combined with our previous studies, these observations suggested that FLP ointment inhibited tumour growth in Lewis lung xenografted mice.

To characterise the exosomes, CD63 and Alix proteins were measured in the serum. Exosomes are released by a variety of cells, such as tumour cells and immune cells [17]. The tumour-derived exosomes (TDEs) secreted by tumour cells contain various proteins, miRNA, DNA, and other components, which participate in the communication between cells [18]. Exosomes can enter the lymphatic system and capillaries within the tumour tissue and play roles in immune evasion and the regulation of T cells and NK cells [19, 20]. The exosomes interact with tumour suppressors and participate in the regulation of tumour growth [21]. An increasing number of studies have noted that exosomes provide stability and transport proteins and nucleic acids into specific target cells, thus playing a critical role in various aspects of the formation of the tumour microenvironment, invasion, metastasis, and angiogenesis [11, 22]. The characteristically expressed exosome proteins were TSG101, Alix, CD9, and CD63. In this study, we examined the expression of CD63 and Alix proteins in the NC-EXOs, MO-EXOs, and FLP-EXOs, which expressed these proteins. The present study found that FLP ointment could increase CD63 and Alix protein levels compared to those of MO mice, which illustrated that FLP ointment could interfere exosomes in serum. FLP ointment may increase the effects of signal transmission through the high levels of exosomes in serum.

The endothelial protein CD31, expressed in vascular endothelial cells, could be considered as a potential marker of microangiogenesis [23]. The present study showed that FLP could downregulate the CD31 expression in tumour tissues and this indicated that FLP could reduce neoangiogenesis. As angiogenesis was restricted by FLP treatment, VEGF and PDGF levels in the serum, exosomes, lung, and tumour tissues were measured in the present study.

Angiogenesis is mainly regulated by several different growth factors. The VEGF family and its receptors are likely to be the most important tissue factors responsible for angioblast differentiation and lymphangiogenesis, which activates the tyrosine kinase receptors of endothelial cells and induces endothelial cell growth and invasion [24, 25]. PDGF and its receptor play an important regulatory role in tumour angiogenesis, stimulating vascular endothelial cell growth and chemotaxis and contributing to tumour angiogenesis and remodelling [26, 27]. A previous study found that FLP ointment inhibited angiogenesis and inhibited the expression of VEGF and PDGFR proteins in the lung tissues [15]. The results of that study agreed with our IHC and WB analyses. Treatment with FLP significantly decreased VEGF, PDGF, and PDGFR levels in lung and tumour tissues. In this study, we found that the levels of VEGF, PDGF, and PDGFR in the serum were increased in mice with lung carcinoma xenografts compared to those of NC mice. FLP ointment decreased VEGF, PDGF, and PDGFR protein expression in the serum compared to that of MO mice. Research has shown that exosomes secreted by HepG2 cells activated a receptor for immune cells and promoted human umbilical vein endothelial cell angiogenesis through the transfer of miRNAs [28]. Upon incubation with exosomes from celecoxib-treated lung cancer cells, the levels of VEGF produced by THP-1 cells were increased [29]. Meanwhile, the expression levels of VEGF, PDGF, and PDGFR in the serum exosomes of Lewis lung carcinoma xenografted mice were significantly increased compared with those of normal mice. CTX and FLP ointment both individually decreased VEGF, PDGF, and PDGFR expression. This result implies that FLP ointment inhibited angiogenesis.

In the process of tumourigenesis and progression, chronic inflammation has become a recognised risk factor for epithelial-derived carcinoma. The environment is composed of tumour cells, interstitial cells, immune cells, cytokines, etc., which make up a dynamic microenvironment called the tumour microenvironment (TME) [30]. The TME is closely related to angiogenesis, which mediates the response to antiangiogenesis drugs and increases the risk of tumour recurrence and metastasis after antiangiogenic therapy [31, 32]. Cancerous cells can influence the TME by releasing extracellular signals, promoting tumour angiogenesis, and inducing peripheral immune tolerance, while the TME can affect the proliferation and migration of cancerous cells [33–35]. Inflammatory cytokines play a key role in shaping the TME [36]. This study examined cytokine levels and found that the levels of inflammation-related cytokines, such as IL-6, IL-1*β*, and TNF-*α*, were decreased in FLP-treated MO mice in the tumour tissues compared to those of MO mice. These results were in accordance with previous studies, which showed that FLP ointment could decrease the expressions of IL-6, IL-1*β*, and TNF-*α* in the serum. Inflammatory cytokines of IL-6 family including IL-6 have been implicated in the migration and invasiveness of human lung carcinoma. And VEGF as a potent angiogenic vascular sprouting could be promote by IL-6 via activation of transcription factors HIF1*α* and STAT3 (gp130 Janus-activated kinase/signal transducer and activator of transcription 3 pathway) [37–40]. IL-1*β* and TNF-*α* are another two inflammatory mediators that could activate their responsive receptors (gp130 and TNFR1) to be involved the NF-*κ*B transcription factor [39, 41]. NF-*κ*B activates VEGF gene expression binding to NF-*κ*B sites in the promoter regions of VEGF gene. Inhibitors and activators of NF-*κ*B could decrease and increase VEGF levels in carcinoma, respectively [42]. TGF-*β*, one role in the pathogenesis of carcinoma, could regulate VEGF release in an ALK-5-dependent manner by SMAD2/3 versus SMAD1/5/8 signalling [43]. In this study, inflammatory cytokines that could enhance the tumourigenic process such as IL-6, IL-1*β*, and TNF-*α* were decreased in FLP-treated MO mice in the tumour tissues and serum compared to those of MO mice, which is in accordance with previous studies. Furthermore, the present study analysed the levels of IL-6, IL-1*β*, and TNF-*α* in exosomes. The expression levels of IL-1*β* and TNF-*α* were significantly increased in exosomes of MO mice compared to those of NC mice, while CTX and FLP ointment could markedly reduce the levels of IL-6, IL-1*β*, and TNF-*α* compared to those of MO mice. This indicated that FLP ointment significantly decreased the expression of IL-6, IL-1*β*, and TNF-*α* in exosomes, serum, lung, and tumour tissues. These results gave a clue that FLP could downregulate tumour inflammatory cytokines levels to lessen the tumourigenic process.

The chemical structures and Mass Spectrogram of ginsenoside Rg3, liquiritin, calycosin, astragaloside VI, and formononetin were shown in Figure 1. Many of the herbal medicine components in FLP ointment have antitumour effects. For example, ginsenoside Rg3 (Radix Panacis Quinquefolii) inhibits tumour migration and invasion through the PI3K-AKT signalling pathway [44] and may improve the sensitivity to cisplatin during lung cancer therapy [45]; liquiritin (Radix Glycyrrhizae) induces apoptotic cell death by upregulating p53 and p21 in A549 non–small cell lung cancer cells [46]; and calycosin, astragaloside VI, and formononetin (Radix Astragali) suppress A549 cell proliferation and migration through the induction of cell cycle arrest and apoptosis via the PKC-*α*/ERK1/2 pathway and the modulation of macrophage polarization through AMPK signalling [47–49]. Thus, these components are the basis for the inhibitory functions in lung cancer.

## 5. Conclusions

The present study suggested that FLP could inhibit tumourigenesis in a Lewis lung xenograft mouse model. Downregulation of tumour inflammatory cytokines and VEGF levels in exosomes, serum, and tumour tissues were involved in carcinoma inhibition. The limitation of this study was that angiogenesis and inflammation indicators had been just detected and following studies will help to investigate the relationship between angiogenesis factors and inflammation cytokines, and the mechanisms of FLP on antiangiogenesis with lung carcinoma should be studied more deeply.

## Figures and Tables

**Table 1:** Chinese medicines contained in FLP ointment.

Chinese name	Latin name	Family	Part used	Voucher codes
Huang qi	Radix Astragali	*Astragalus membranaceus* (Fisch.) Bge. var. *mongholicus* (Bge.) Hsiao	Root	20170927
Xi-Yang-Shen	Radix Panacis Quinquefolii	*Panax quinquefolius* L.	Root	20170922
Mai-Dong	Radix Ophiopogonis	*Ophiopogon japonicas* (Thunb.) Ker-Gawl.	Root	20171118
Bei-Sha-Shen	Radix Glehniae	*Glehnia littoralis* Fr. Schmidt ex Miq.	Root	20171025
Xian-He-Cao	Herba Agrimoniae	*Agrimonia pilosa* Ledeb.	Herb	20171102
Quan-Shen	Rhizoma Bistortae	*Polygonum bistorta* L.	Root	20170904
Bai-Jiang-Cao	Herba Patriniae	*Patrinia villosa* (Thunb.) Juss.	Herb	20170905
San-qi	Radix Notoginseng	*Panax notoginseng* (Burk.) F. H. Chen	Root	20171105
Chuan-Bei-Mu	Bulbus Fritillariae Cirrhosae	*Fritillaria cirrhosa* D. Don	Bulb	20170921
Gan-Cao	Radix Glycyrrhizae	*Glycyrrhiza uralensis* Fisch.	Root	20170925
Dong-Cong-Xia-Cao	Cordyceps	*Cordyceps sinensis* (Berk.) Sacc.	Stroma & Larva	20171024
Tao-Ren	Semen Persicae	*Prunus persica* (L.) Batsch	Fruit	20171012
Ku-Xing-Ren	Semen Armeniacae Amarum	*Prunus armeniaca* L. var. *ansu* Maxim.	Fruit	20171019

**Table 2:** Components of FLP ointment identified by UHPLC-Orbitrap MS.

No.	*t*_R_	Precursor ion (*m*/*z*)		Diff. (ppm)	Elem. Comp.	Identification
1	1.27	[M + Na]^+^	365.1047	−2.061	C_12_H_22_O_11_Na	Maltose
2	1.27	[M + Na]^+^	365.1047	−1.946	C_18_H_32_O_16_Na	D-Melezitose
3	1.27	[M + Na]^+^	509.1468	−1.829	C_18_H_30_O_15_Na	Beta-glucan
4	1.35	[M + H]^+^	305.1333	−3.269	C_12_H_21_N_2_O_7_	2′-Deoxymugineic acid
5	2.59	[M + H]^+^	145.0493	−1.898	C_6_H_9_O_4_	Monoethyl fumarate
6	3.21	[M + H]^+^	127.0387	−2.130	C_6_H_7_O_3_	5-Hydroxymethylfurfural
7	4.35	[M + H]^+^	163.0368	−2.150	C_9_H_7_O_3_	Umbelliferone
8	13.74	[M + Na]^+^	447.1277	3.337	C_20_H_24_O_10_Na	Praeroside II
9	13.74	[M + Na]^+^	469.1094	−2.298	C_22_H_22_O_10_Na	Calycosin-7-O-beta-D-glucoside
10	15.31	[M + Na]^+^	463.0862	3.332	C_19_H_20_O_12_Na	4-O-(E)-Caffeoyl-5-O-malonylquinic acid
11	15.84	[M + Na]^+^	491.2454	−1.879	C_21_H_40_O_11_Na	2,6-Dimethyl-4-heptyl-*β*-D-maltopyranoside
12	17.76	[M + Na]^+^	447.0914	−1.695	C_19_H_20_O_11_Na	6-O-galloylarbutin
13	18.18	[M + H]^+^	771.4877	−1.541	C_41_H_71_O_13_	Ginsenoside F3
14	18.29	[M + H]^+^	639.4451	−2.486	C_36_H_63_O_9_	Ginsenoside F1
15	18.40	[M + H]^+^	621.4347	−2.261	C_36_H_61_O_8_	Ginsenoside Rh4
16	19.47	[M + H]^+^	639.4455	−1.814	C_36_H_63_O_9_	Ginsenoside Rh1
17	19.47	[M + H]^+^	785.5029	−2.162	C_42_H_73_O_13_	Ginsenoside Rg3
18	19.79	[M + H]^+^	419.1329	−1.810	C_21_H_23_O_9_	Liquiritin
19	19.90	[M + H]^+^	489.1383		C_24_H_25_O_11_	Kaempferide, 7-O-(4-O-acetyl-*α*-L-rhamnopyranosyl)
20	20.01	[M + H]^+^	431.1328	−2.038	C_22_H_23_O_9_	Ononin
21	20.97	[M + Na]^+^	471.2195	−1.249	C_21_H_36_O_10_Na	Geranyl b-primeveroside
22	20.97	[M + Na]^+^	823.4806	−1.017	C_42_H_72_O_14_Na	Ginsenoside Rg1
23	21.19	[M + Na]^+^	969.5375	−1.853	C_48_H_82_O_18_Na	Ginsenoside Re
24	21.94	[M + Na]^+^	485.1410	−1.707	C_23_H_26_O_10_Na	Astrapterocarpan
25	22.15	[M + H]^+^	285.0752	−1.824	C_16_H_13_O_5_	Calycosin
26	22.25	[M + H]^+^	167.0700	−1.860	C_9_H_11_O_3_	(R)-(-)-methyl mandelate
27	22.56	[M + H]^+^	539.1852	−2.094	C_28_H_33_O_14_	Luteolin-7-O-rutinoside
28	22.77	[M + H]^+^	1007.5046	−1.161	C_48_H_78_O_22_	Aesculuside B
29	22.99	[M + Na]^+^	487.1565	−1.946	C_23_H_28_O_10_Na	7,2′-Dihydroxy-3′,4′-dimethoxyisoflavane-7-O-glucoside
30	24.70	[M + H]^+^	1093.5400	−2.277	C_52_H_85_O_24_	Deapioplatycodin D
31	25.35	[M + H]^+^	845.4517	−1.498	C_42_H_69_O_17_	Saniculoside A
32	25.88	[M + H]^+^	639.4456	−1.626	C_36_H_63_O_9_	Sanchinoside B1
33	27.17	[M + H]^+^	947.5196	−1.495	C_47_H_79_O_19_	Astragaloside VI
34	27.81	[M + H]^+^	801.4976	−2.399	C_42_H_73_O_14_	Ginsenoside Rg1
35	28.55	[M + H]^+^	855.3994	−1.802	C_42_H_63_O_18_	22-Hydroxyl-licorice-saponin G2
36	28.55	[M + H]^+^	423.3216	−1.774	C_30_H_45_O_5_	(R)-4,4,6a,6b,8a,11,11,14b-Octamethyl-1,2,3,4,4a,5,6,6a,6b,8a,9,10,11,12,12a,14b-hexadecahydro-picen-3-ol
37	28.77	[M + H]^+^	683.3992	−1.366	C_36_H_59_O_12_	3-O-*β*-glucosyl-platycodigenin
38	28.98	[M + H]^+^	315.1221	−1.905	C_18_H_19_O_5_	Agrimonolide
39	28.98	[M + H]^+^	477.1745	−2.208	C_24_H_29_O_10_	Agrimonolide-6-O-glucopyranoside
40	29.19	[M + Na]^+^	1131.5902	−1.723	C_54_H_92_O_23_Na	Ginsenoside Rb1
41	29.40	[M + H]^+^	269.0804	−1.469	C_16_H_13_O_4_	Formononetin
42	30.45	[M + Na]^+^	807.4486	−1.880	C_41_H_68_O_14_Na	Astragaloside III
43	31.40	[M + H]^+^	839.4070	−1.569	C_42_H_63_O_17_	22-Hydroxyl-glycyrrhizin
44	31.82	[M + Na]^+^	849.4584	−2.734	C_43_H_70_O_15_Na	Isoastragalosides II
45	32.34	[M + H]^+^	823.4097	−1.618	C_46_H_63_O_16_	Glycyrrhizinic acid
46	32.34	[M + H]^+^	647.3776	−2.092	C_36_H_54_O_10_	Glycyrrhizinic acid 3-O-glucuronide
47	33.07	[M + H]^+^	785.5032	−1.704	C_42_H_73_O_13_	Ginsenoside Rg3
48	35.26	[M + H]^+^	647.3779	−1.706	C_36_H_55_O_10_	Glycyrrhetinic acid monoglucuronide
49	35.26	[M + Na]^+^	891.4695	−1.926	C_45_H_72_O_16_Na	Isoastragaloside I
50	38.36	[M + H]^+^	425.3771	−1.605	C_30_H_49_O	Pachysandienol-A
51	38.36	[M + Na]^+^	789.4744	−2.000	C_42_H_70_O_12_Na	Ginsenoside Rg5
52	39.72	[M + H]^+^	277.1795	−1.050	C_17_H_25_O_3_	Cyclandelate
53	40.23	[M + H]^+^	471.3463	−1.159	C_30_H_47_O_4_	Glycyrrhetinate

## Data Availability

The data used to support the findings of this study are available from the corresponding author upon request.

## Supplementary Materials

Supplementary Materials Identification of compounds in FLP ointment.
